# Neural Progenitor Cells Promote Axonal Growth and Alter Axonal mRNA Localization in Adult Neurons

**DOI:** 10.1523/ENEURO.0171-16.2017

**Published:** 2017-02-03

**Authors:** Tanuja T. Merianda, Ying Jin, Ashley L. Kalinski, Pabitra K. Sahoo, Itzhak Fischer, Jeffery L. Twiss

**Affiliations:** 1 Department of Anatomy and Neurobiology, Drexel University College of Medicine, Philadelphia, Pennsylvania 19129; 2 Department of Biological Sciences, University of South Carolina, Columbia, South Carolina 29208

**Keywords:** axonal transport, glial restricted progenitor, mRNA localization, neural restricted progenitor, stem cell

## Abstract

The inhibitory environment of the spinal cord and the intrinsic properties of neurons prevent regeneration of axons following CNS injury. However, both ascending and descending axons of the injured spinal cord have been shown to regenerate into grafts of embryonic neural progenitor cells (NPCs). Previous studies have shown that grafts composed of glial-restricted progenitors (GRPs) and neural-restricted progenitors (NRPs) can provide a permissive microenvironment for axon growth. We have used cocultures of adult rat dorsal root ganglion (DRG) neurons together with NPCs, which have shown significant enhancement of axon growth by embryonic rat GRP and GRPs/NRPs, both in coculture conditions and when DRGs are exposed to conditioned medium from the NPC cultures. This growth-promoting effect of NPC-conditioned medium was also seen in injury-conditioned neurons. DRGs cocultured with GRPs/NRPs showed altered expression of regeneration-associated genes at transcriptional and post-transcriptional levels. We found that levels of GAP-43 mRNA increased in DRG cell bodies and axons. However, hepcidin antimicrobial peptide (HAMP) mRNA decreased in the cell bodies of DRGs cocultured with GRPs/NRPs, which is distinct from the increase in cell body HAMP mRNA levels seen in DRGs after injury conditioning. Endogenous GAP-43 and β-actin mRNAs as well as reporter RNAs carrying axonally localizing 3'UTRs of these transcripts showed significantly increased levels in distal axons in the DRGs cocultured with GRPs/NRPs. These results indicate that axon growth promoted by NPCs is associated not only with enhanced transcription of growth-associated genes but also can increase localization of some mRNAs into growing axons.

## Significance Statement

Transplants of neural progenitor cells (NPCs) into the injured spinal cord have been used to allow the formation of a “relay circuit” that could restore functional connections across severed spinal tracts. To establish this relay, host axons from the injured spinal cord must grow into the graft/injury site and form synaptic connections with graft-derived neurons. Our study indicates that NPCs can alter the expression of regeneration-associated genes in adult sensory neurons at both the transcriptional and post-transcriptional levels. The post-transcriptional changes are manifested by increased axonal localization of regeneration-associated mRNAs, which likely contributes to the increased growth capacity of DRG neurons by local protein synthesis.

## Introduction

Transplantation of neural progenitor cells (NPCs) has long been regarded as a potential strategy to replace or repair cellular components that are lost when the nervous system has been injured or is diseased (Limke and Rao, 2002; Vishwakarma et al., 2014; Fortin et al., 2016). The utility of such cell-replacement therapies requires that the cells produced by the transplants integrate into the host nervous system. Remarkable advances have been made for grafting various types of NPCs into the adult spinal cord after experimental injury to produce oligodendrocytes, astrocytes, and neurons, and the NPC grafts show promising therapeutic effects in some instances. For example, grafting embryonic NPCs composed of neuronal-restricted progenitors (NRPs) and glial-restricted progenitors (GRPs) into the injured spinal cord allowed the production and integration of new neurons, which receive synaptic input from host neurons and extend axons through the typically nonpermissive environment of the adult spinal cord (Dulin and Lu, 2014; Lane et al., 2016). Robust axon growth from the neurons derived from these embryonic progenitors is an exciting contrast to the typically failed regenerative growth of endogenous axons after spinal cord injury. Following host synaptic connections with progenitor-derived neurons, functional recovery after spinal cord injury could be achieved through a relay circuit if axons of the progenitor-derived neurons reach and synapse with appropriate targets in the host spinal cord. Both the Fischer and Tuszynski groups have demonstrated such a relay circuit by grafting progenitor cells from the embryonic spinal cord into the spinal cord of adult rodents in sensory and motor systems (Bonner et al., 2011; Lu et al., 2012).

Neurons derived from these grafted progenitor cells have properties of embryonic neurons with high growth capacity as well as the ability to grow on nonpermissive CNS substrates. Indeed, neurons derived from cultures of GRP/NRP isolated from embryonic day 13.5 (E13.5) spinal cord are able to extend axons onto nonpermissive substrates [e.g., chondroitin sulfate proteoglycan (CSPG)] that normally attenuate axon growth of more mature neurons (Ketschek et al., 2012). Although the regeneration capacity of injured spinal cord axons is normally quite low, it is likely that the grafted progenitor cells promote axon growth from adult neurons in the host spinal cord through the production of permissive astrocytes by the GRP. The supportive properties of GRPs in promoting axon growth and regeneration have been demonstrated both *in vitro* and *in vivo*, including the role they play in neuronal survival and differentiation and the formation of functional synapses (Lepore and Fischer, 2005; Bonner et al., 2011; Jin et al., 2011; Ketschek et al., 2012; Haas and Fischer, 2014).

Since NPCs have the potential to modulate the growth of host axons, it is possible that grafted precursor cells can regulate the expression of regeneration-associated genes in the mature neurons of the host. In the peripheral nervous system (PNS), where injured axons can spontaneously regenerate, axotomy changes neuronal gene expression programs to support axon growth (Perry and Fainzilber, 2014). Successful regeneration of injured axons requires a coordination of these gene expression changes with localized mechanisms in axons that are needed for axon extension. Studies over the last 2 decades have shown that localized translation of mRNAs in axons contributes to PNS axon regeneration. The translation of Importin β1 mRNA in axons results in a retrograde signal that changes gene expression programs after injury (Perry et al., 2012). Mice with decreased axonal localization of GAP-43 and β-actin mRNAs, from knockout or depletion of the RNA binding protein ZBP1 (also called IGFII mRNA binding protein 1), show delayed regeneration of PNS axons (Donnelly et al., 2011). A few publications have further indicated that injured and regenerating axons in the mature spinal cord may translate proteins (Willis et al., 2011; Walker et al., 2012; Kalinski et al., 2015). So this intra-axonal protein synthesis could contribute to axon growth in the mature CNS. However, axonal protein synthesis has also been detected in response to capsaicin in the PNS, and neurotransmitters and amyloid β peptides in the CNS (Baleriola and Hengst, 2015; Hsu et al., 2015), indicating that the functions of axonally generated proteins clearly go beyond the support of axon growth.

Here, we have begun to address the potential mechanisms of axon growth that grafts of GRPs/NRPs might have in host axons. We show that GRPs and GRPs/NRPs increase the growth of axons from sensory neurons cultured from the adult dorsal root ganglion (DRG) either in coculture experiments or by the use of conditioned media. Importantly, the adult DRG neurons cocultured with these NPCs show alterations in the expression of growth-associated genes and in axonal levels of several mRNAs compared with DRGs cultured under standard conditions. Since localized translation of several gene products has been linked to increases in axon growth (Perry and Fainzilber, 2014), the post-transcriptional alterations seen here in host sensory neurons likely support the growth of these axons.

## Materials and Methods

### Animal care and use

All animal experiments were approved by institutional animal care and use committees. Male and female Sprague Dawley rats (weight, 150-225 g) were used for the culturing of DRGs. No differential responses were noted in DRGs harvested from male versus female animals in the studies below. For injury conditioning, rats were subjected to sciatic nerve crush at mid-thigh, as previously described (Twiss et al., 2000). Seven days after nerve crush, animals were killed and L4/5 DRGs were isolated for dissociated culture preparations.

Progenitor cells were isolated from spinal cords of rat embryos (E13.5) as previously described (Bonner et al., 2011). Briefly, embryos were placed in DMEM/F-12 and the meninges removed by incubation in collagenase I (10 mg/ml; Worthington) plus dispase II (20 ng/ml; Worthington) in HBSS for 9 min at room temperature. Spinal cords then were then treated with trypsin (0.5%)/EDTA for 20 min at 37°C, dissociated, and plated as outlined below.

### Cell culture

Dissociated E13.5 spinal cord progenitor cells were plated on poly-l-lysine (PLL; 15 µg/ml; Sigma-Aldrich) and laminin (15 µg/ml; Invitrogen) in basal medium composed of DMEM/F12 (Invitrogen), BSA (1 mg/ml; Sigma-Aldrich), B27 (20 μl/ml; Invitrogen), penicillin/streptomycin solution (50 IU/ml; Invitrogen), 1 × N2 supplement (Invitrogen), that was supplemented with bFGF and NT-3 (20 and 10 ng/ml, respectively; PeproTech). To enrich for GRP, progenitor cells were cultured for 10 d on PLL/laminin-coated culture dishes in GRP–basal medium supplemented with 20 µg/ml bFGF prior to the freezing of cultures at 2 × 10^6^ cells/ml in freezing medium (80% GRP basal medium supplemented with 10 ng/ml bFGF, 10% Chick Embryo Extract, and 10% DMSO) at −80°C (Haas et al., 2012). In all experiments, GRP and GRP/NRP cells were used by the third passage.

Cultures of embryonic rat GRPs and mixed GRPs/NRPs were generated from frozen aliquots as previously described (Haas et al., 2012; Hayakawa et al., 2015). GRP or GRP/NRP cell mixtures were plated at a concentration of 10,000 cells/190 mm^2^ in GRP–basal medium supplemented with 10 ng/ml bFGF for expansion. At least 24 h prior to experiments for coculture or collecting conditioned medium, growth medium was aspirated, and cultures were rinsed with DMEM/F12 and then incubated in GRP–basal medium without any FGF or NT3 added. Conditioned medium was collected from GRP or GRP/NRP cultures 48 h after plating, with matched cell densities across the replicated experiments (Ketschek et al., 2012). The conditioned medium was transferred to DRG cultures 24 h after plating the DRGs (see below); axon outgrowth from DRGs was analyzed 48 h later [3 d *in vitro* (DIV) for DRGs].

For coculture experiments, GRPs, GRPs/NRPs, and dissociated DRGs were plated onto PLL/laminin-coated glass coverslips or tissue culture plates. For explant DRG cocultures, polyethylene-terephthalate (PET) membrane tissue culture inserts (3 μm pores; Falcon) were coated with 100 μg/ml PLL and 6.5 μg/ml laminin.

For cultures of adult sensory neurons, DRGs were dissociated using 1% collagenase (365 U/mg type I; Worthington) for 1.5 h followed by 1 × trypsin (Sigma-Aldrich) for 30 min at 37°C, 5% CO_2_. After trituration, DRGs were pelleted at 100 × *g* for 20 min and then washed with DMEM/F12 supplemented with 15% BSA. After two additional washes, dissociated ganglia were plated onto coated glass coverslips. For coculture with GRP or GRP/NRP, dissociated DRGs were plated onto a bed of progenitors that had been plated 1 d previously; coverslips were processed identically without GRP or NRP/GRP for control. To evaluate axonal growth from injury-conditioned DRG neurons, dissociated L4/5 DRGs were plated at low density on PLL/laminin-coated coverslips that were placed on top of a bed of NRP/GRPs. Coverslips were separated from NRPs/GRPs using beads of paraffin on the under surface of coverslips. For control, injury-conditioned DRGs were processed and plated in an identical fashion, except for the coverslips, with paraffin beads and laid onto a PLL/laminin-coated plate. In both cases, GRP basal medium was used (replaced at the time of DRG culturing). The culture medium for these experiments consisted of the basal–GRP medium (bFGF and NT3 excluded), and cultures were incubated at 37°C, 5% CO_2_. For analyses of conditioned medium effects, DRGs were plated into GRP–basal medium on PLL/laminin-coated coverslips, and then the medium was replaced the following morning with GRP–basal medium that had been exposed to GRP or GRP/NRP cultures for 24 h. For control, DRGs were exposed to GRP–basal medium that had been similarly incubated at 37°C, 5% CO_2_ for equivalent duration.

For explant cultures, ganglia were rinsed in medium containing antibiotics, as above, and then directly plated onto tissue culture inserts (4-5 DRGs/insert). The explants were initially cultured in DMEM/F12, 1 × N1 supplement (Sigma-Aldrich), 10% horse serum (Hyclone), and 10 μm cytosine arabinoside (Sigma-Aldrich) at 37°C, 5% CO_2_. After 3 DIV, the membrane inserts were transferred to wells with GRP/NRP that had been plated 2 d previously in GRP–basal medium, as cited above. Membrane inserts were either fixed for immunostaining or used for isolation of the cell body or axonal RNA from upper and lower membrane surfaces after 3 d of exposure to progenitor cells (6 DIV total).

### Plasmids, viral preparations, and transfections

Diffusion limited eGFP^MYR^ reporter construct with 5'UTR of CamKIIα (5'camkII) and various 3'UTRs of β-actin, γ-actin, calreticulin (calr), and GAP43 have been published previously (eGFP^MYR^3'β-actin, eGFP^MYR^3'γ-actin, eGFP^MYR^3'calr, eGFP^MYR^3'gap43, respectively; Willis et al., 2007; Vuppalanchi et al., 2010; Yoo et al., 2013). For transfection, dissociated DRGs were resuspended in transfection solution (Basic Neuron SCN Kit, Lonza) along with 3 μg of plasmid DNA; dissociated DRGs were then transfected using program 8 of the AMAXA Nucleofector apparatus (Lonza). Cells were pelleted at 100 × *g*, resuspended in basal medium, and plated on PLL/laminin-coated coverslips alone or with a bed of GRPs/NRPs, as above. Medium was replaced after 24 h, and cultures were fixed after 72 h and processed for fluorescence *in situ* hybridization (FISH) combined with immunofluorescence (IF; see below).

For explant cultures, transduction with AAV5 encoding a mCherry^MYR^ with axonally localizing 3'UTR of rat high mobility group box 1 (HMGB1)/amphoterin (amph) mRNA (mCh^MYR^3'amph; Merianda et al., 2015) was used to further visualize cell bodies and axons. For this, ganglia were incubated in 25 µl of basal medium containing 1 × 10^9^ viral particles at 37°C, 5% CO_2_ for 30 min prior to plating onto tissue culture inserts.

### RNA isolation and analyses

DRG explant cultures on PET inserts were used for harvesting RNA from axon and cell body compartments (Willis et al., 2005). For axons, the bottom of the membrane was scraped with a cotton-tipped applicator, and RNA was isolated from the scraped axons using the RNAqueous^micro^ kit (Ambion) per the manufacturer specifications. Ganglia were removed from the upper surface of the membrane using jeweler forceps, and RNA was isolated using the RNAqueous kit (Ambion) per manufacturer specifications. RNA isolates were quantified using a VersaFluor fluorimeter (Bio-Rad) with RiboGreen reagent (Invitrogen). Equal quantities of RNA from cell body or axons were used as a template for reverse transcription (RT) with iSCRIPT (Bio-Rad). RT reactions were diluted 10-fold, and axonal isolates were tested for enrichment by RT-PCR for β-actin, MAP2, c-Jun, and GFAP mRNAs with HotStarTaq (Qiagen; Merianda et al., 2013). Negative control for amplification consisted of RNA processed without the addition of RT.

Droplet digital PCR (ddPCR) was used to assess mRNA levels in RT reactions from axon versus cell body compartment RNAs. For this, the equivalent of 0.01–3.0 ng of RNA from was diluted from the RT reactions and used as template for transcript-specific amplification using Taqman primer and probe sets (Integrated DNA Tech; sequences available on request) and ddPCR Supermix per manufacturer protocol (Bio-Rad). Amplification products were read on a QX200 Droplet Digital instrument (Bio-Rad). The input RNA mass for ddPCR was optimized for each primer set to ensure that there was no saturation of droplets. HMGB1/amphoterin mRNA was previously shown to be constitutively transported into adult DRG axons (Merianda et al., 2015), so each ddPCR reaction was duplexed with HMGB1/amphoterin primers plus probe as a loading control. All analyses were performed on at least three biological replicates.

### *In situ* hybridization and immunofluorescence

To detect endogenous mRNAs by FISH, Cy3-labeled antisense “Stellaris” DNA oligonucleotide probes were purchased from LGC BioSearch Technologies. Custom probes were designed using the Stellaris Probe Designer web tool from LGC Biosearch Technnologies (www.biosearchtech.com/support/tools). Cy3-labeled, scrambled oligonucleotides were used to control for specificity. All probe sequences are available upon request from the authors. *In situ* hybridization, washes, and IF testing were performed as described previously (Spillane et al., 2013). Primary antibodies consisted of a cocktail of chick anti-neurofilament light (NFL), anti-neurofilament medium (NFM), and anti-neurofilament heavy (NFH) antibodies (Aves; 1:200), and secondary antibodies consisted of FITC-conjugated anti-IgY (1:400; Jackson ImmunoResearch).

GFP mRNA was detected in the transfected DRGs using digoxigenin-labeled cRNA probes for FISH as described previously (Merianda et al., 2013). Sense and antisense cRNA probes were generated by *in vitro* transcription with SP6 or T7 RNA polymerases from linearized pcDNA3-eGFP (Addgene). After fixation, permeabilization, and prehybridization at room temperature, coverslips were hybridized at 55°C for 18 h with 5 ng/ml antisense or sense cRNA probes. After washing, coverslips were processed for IF, as described previously (Merianda et al., 2013), using rabbit anti-βIII-tubulin antibody (1:500; Millipore) followed by aminomethylcoumarin (AMCA)-conjugated goat anti-rabbit (1:400; Jackson ImmunoResearch) and Cy3-conjugated mouse anti-digoxigenin antibodies (1:200; Jackson ImmunoResearch). Coverslips for both Stellaris and cRNA probe FISH/IF were mounted with Prolong Gold Antifade (Invitrogen). RNA signal intensities in cell body and distal axons were analyzed for at least 20 neurons per condition over at least three separate transfection experiments.

Standard IF on coverslips and PET membranes was performed as previously published (Merianda et al., 2015). Primary antibodies consisted of rabbit anti-βIII-tubulin (1:500; Covance), mouse anti-alkaline phosphatase (1:400; Novus), and mouse anti-human nuclear antigen (1:400; Millipore). Secondary antibodies were AMCA- or FITC-conjugated anti-mouse and anti-rabbit IgGs (1:200 for each; Jackson ImmunoRes.). For staining rat progenitor cells cocultured with explant DRGs, the GRPs/NRPs on coverslips were stained with Vybrant DiO cell-labeling solution per manufacturer recommendations (Molecular Probes).

RNA and protein signals in the dissociated DRG cultures were captured using Olympus (GFP mRNAs) or Leica epifluorescent (endogenous mRNAs) microscopes fitted with a CCD camera. For comparing signal intensities in FISH/IF experiments, digital images matched for exposure time, gain, offset, and postprocessing parameters were used. βIII-tubulin or neurofilament immunoreactivity and differential interference contrast images were used to trace neuronal cell body and terminal axons in these experiments. ImageJ was then used to calculate the average number of pixels per square micrometer in these areas, as described previously (Merianda et al., 2013). Regions of interest for these axonal analyses consisted of a 100 µm segment of axon shaft separated from the cell body by at least 400 µm.

Leica SP8 confocal microscope was used to image explant DRGs cocultured with GRPs/NRPs. For this, the PET membrane with explants was excised after IF and imaged in PBS using a chambered coverslip with a weighted harp to avoid shifting of the membrane. DiO-stained GRPs/NRPs were imaged separately.

### Axon outgrowth assays

Neurite outgrowth from DRG-GRP and DRG-GRP/NRP cocultures and DRGs treated with conditioned medium was analyzed from random images of βIII-tubulin-immunostained coverslips. The length of the longest axon of individual images was measured by using ImageJ, as described previously (Donnelly et al., 2011). For dissociated DRGs plated on coverslips over a bed of GRPs/NRPs, neurite growth was analyzed using the WIS-Neuromath software package (Rishal et al., 2013) from cultures stained for NFL, NFM, and NFH. For this, an automated stage was used to capture all neurons on 12 mm coverslip for analyses. At least three separate culture preparations were analyzed for each condition.

### Statistical analyses

The Prism 4 software package (GraphPad) was used for all statistical analyses. The Student’s *t* test was used to compare two means of independent groups in the axonal growth assays and fluorescence intensity comparisons from FISH/IF images.

## Results

### Glial-restricted progenitor cells increase neurite outgrowth from adult DRG neurons

**Figure 1.**
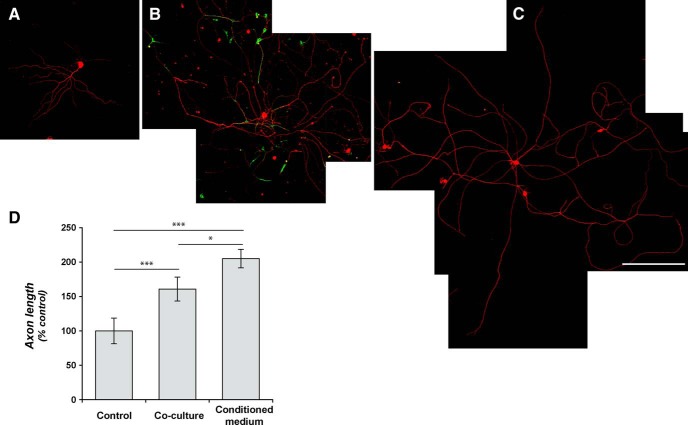
GRPs enhance axonal growth in DRGs *in vitro*. ***A–C***, Representative montage images are shown for control dissociated DRG culture (***A***), dissociated DRGs cocultured with rat GRPs (***B***), and dissociated DRGs cultures exposed to conditioned medium from alkaline phosphatase-expressing rat GRPs (***C***). βIII-tubulin (red) immunofluorescence highlights the neurons; GRPs are visualized by immunostaining for alkaline phosphatase (green). ***D*** shows quantification for the average length of the longest axon per neuron ± SEM (*n* ≥ 30 neurons in three separate experiments; **p ≤ 0.01 by Student’s *t* test). Scale bar, 250 μm.

Transplantation of NPCs derived from embryonic spinal cord supports the growth of host sensory axons into the grafted progenitor cells following spinal cord injury (Bonner et al., 2010, 2011; Lu et al., 2012). Conditioned medium from high-density, but not low-density, GRP cultures was also shown to increase the ability of axons to grow into nonpermissive CSPG substrates, both for embryonic chick and rat DRGs (Ketschek et al., 2012). DRG neurons spontaneously extend axonal-like processes in culture, even for neurons harvested from adult rodents (Smith and Skene, 1997). Several different stimuli have been shown to increase or decrease this neurite outgrowth. Here, we started with a minimalist approach to determine whether exposure to embryonic progenitor cells directly impacts mechanisms of axon growth of the adult DRG neurons (Haas et al., 2012; Hayakawa et al., 2015). Dissociated DRGs cocultured on a bed of GRPs grew consistently longer neurites over 48 h *in vitro* compared with dissociated DRGs cultured under standard conditions (Fig. 1, compare *A*, and *B*, *D*). Since conditioned medium from rat GRPs has been shown to increase the ability of embryonic DRGs to grow on nonpermissive substrates (Ketschek et al., 2012), we collected medium from isolated GRP cultures and applied this to dissociated DRG cultures. The GRP-conditioned medium increased neurite outgrowth from the adult DRGs, with neurites significantly longer than the control DRG; the DRG neurites were also significantly longer in cultures treated with the GRP-conditioned medium than those seen in the coculture with GRPs (Fig. 1, compare *C*, *A*; *C*, *B* and *D*). Thus, consistent with a previous report (Ketschek et al., 2012), these data suggest that embryonic GRPs secrete factors that support neurite outgrowth from adult sensory neurons.

### Neurite outgrowth from adult DRG neurons is increased by coculture with glial-restricted progenitor/neural-restricted progenitor mixture

**Figure 2.**
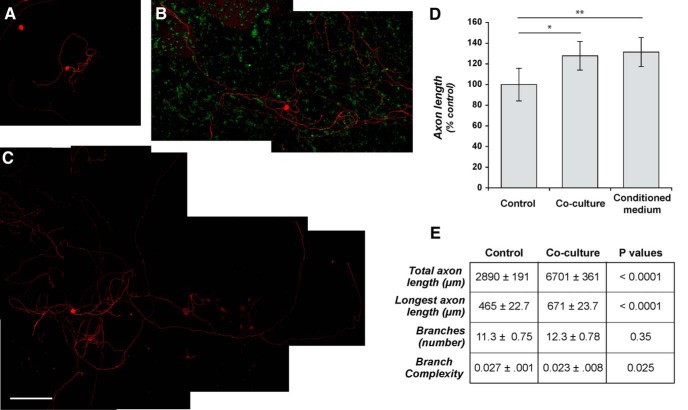
Coculture with neural and glial progenitor cells increases axonal outgrowth from adult DRG neurons. ***A–C***, Representative montage images of control-dissociated DRG cultures (***A***), dissociated DRGs cocultured with rat GRPs/NRPs (***B***), and dissociated DRG cultures exposed to conditioned medium from parallel rat GRP/NRP cultures (***C***) are shown. Immunofluorescence for βIII-tubulin (red) and nestin (green) highlight DRG neurons and GRP/NRP nuclei, respectively. ***D***, Quantitation of the average lengths of the longest axon per neuron (±SEM) for the above conditions is shown. Coculture with GRPs/NRPs significantly increases in axon length compared with the standard DRG culture; exposure to conditioned medium from GRP/NRP cultures showed a further increase in axon length (*n* ≥ 30 neurons in three separate experiments; *p ≤ 0.05 and ***p ≤ 0.001 by Student’s *t* test). Scale bar, 250 μm. ***E***, Quantitation of axon growth parameters for 7 d injury-conditioned DRG neurons cultured on coverslips laid over a bed of GRP/NRP cells (coculture) or control conditions is shown (*n* ≥ 200 neurons analyzed in three separate experiments; *p* values represent ANOVA with Tukey *post hoc* analyses).

Transplants of GRPs along with NRPs into injured spinal cord were shown to result in a relay circuit where host axons synapse with neurons derived from the graft (Bonner et al., 2011). We reasoned that the combination of GRPs/NRPs should similarly stimulate axon growth from the dissociated DRG neurons. Coculture of adult rat DRGs with GRPs/NRPs derived from E13.5 spinal cord-generated DRG neurites significantly longer than control dissociated DRGs cultured in the same medium without progenitor cells (Fig. 2, compare *A*, *B* and *D*). Conditioned medium from the GRP/NRP cocultures also increased neurite outgrowth from the adult DRGs (Fig. 2, compare *C*, *A*). Comparing the cultures treated with GRP/NRP-conditioned medium to the coculture condition, some neurons showed exaggerated neurite growth with the conditioned medium that was similar to what was seen with GRP-conditioned medium (Fig. 2, compare *C*, *B*). However, the growth was not uniform, and the quantitation of neurite lengths showed that DRGs exposed to GRP/NRP-conditioned medium had significantly longer neurites than control DRGs but there was no significant difference comparing DRGs exposed to conditioned medium to those cocultured with GRPs/NRPs (Fig. 2*D*). Thus, both GRP and GRP/NRP cells support neurite outgrowth from adult sensory neurons. Interestingly, the DRG axons appeared to have less contact with GRP/NRP cells than in the GRP cocultures, despite an apparent higher cell density for the GRPs in the GRP–DRG coculture condition (compare Figs. 2*B*, 1*B*). The reason for this is not clear.

DRG neurons that are conditioned by an *in vivo* axotomy several days prior to culture show accelerated axonal growth in culture compared with DRG neurons that have not undergone a preconditioning injury prior to culture (“naive neurons”; Smith and Skene, 1997). Thus, we asked whether the growth-promoting effect of the NPCs seen above is limited to naive neurons or also extends to injury-conditioned DRG neurons. For this, we cultured 7 d injury-conditioned neurons at low density on coverslips over a bed of GRPs/NRPs. Since these injury-conditioned DRG neurons could be cultured at a lower density than the naive DRGs used above, we advanced to an automated approach to assess several axon growth parameters in these cocultures. The injury-conditioned DRG neurons showed significantly longer neurite length, and both the longest axon/neuron and summed total axon length/neuron when exposed to GRPs/NRPs (Fig. 2*E*). Although the number of primary neurites originating from the cell body showed no significant differences from the injury-conditioned DRG neurons on exposure to GRPs/NRPs, the ratio of total branch number to confirmed neurite length (“branching complexity”) calculated by the WIS-Neuromath program showed a small but statistically significant decrease in the injury-conditioned DRGs cocultured with GRPs/NRPs (Fig. 2*E*). Since NPCs effectively increase growth from the injury-conditioned DRGs, these data suggest that the mechanisms of the actions of NPCs upon adult DRG neurons are distinct from the injury-conditioning effect.

### Exposure to GRP/NRP increases gene expression in adult DRG neurons

**Figure 3.**
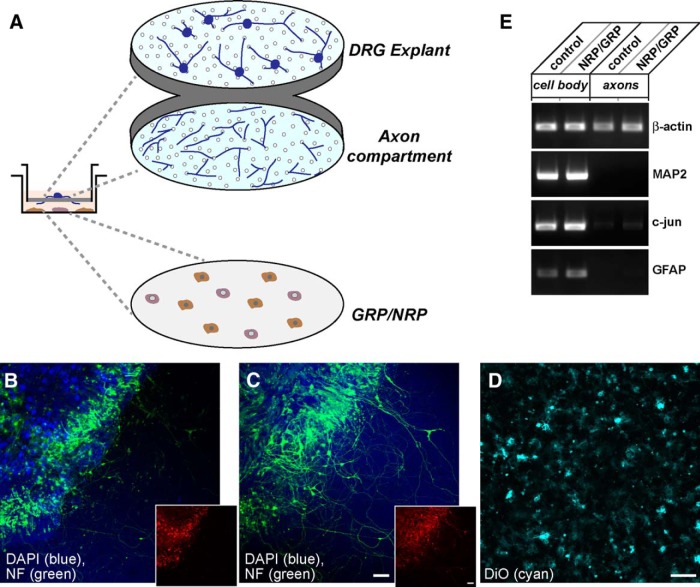
Approach for DRG/progenitor cell coculture to isolate axonal processes. ***A***, Schematic of modified Boyden chamber used for culture system for isolation of axons from DRG neurons is shown. For this, DRG explants were plated onto the upper surface of a porous PET membrane, as previously used for dissociated DRGs (Zheng et al., 2001), and GRPs/NRPs were cultured on the surface of the plate well. ***B–D,***Representative confocal projection images of upper membrane surface with DRGs (***B***), corresponding image stacks for lower membrane surface showing a dense array of axons (***C***), and GRPs/NRPs along the plate surface (***D***) are shown. DRGs were stained for βIII-tubulin (green) and DAPI (blue). The GRPs were visualized with DiO stain (cyan). The insets in ***B*** and ***C*** show mCherry signals (red) of AAV5-mCh^MYR^3'amph-transduced DRGs in cell bodies (***B***) extending into axons along the lower membrane surface (***C***). Scale bars: ***B***, ***C***, 100 µm; ***D***, 50 µm. ***E***, Representative RT-PCR from cell body and axonal isolates for DRGs cultured under control conditions or over a bed of GRPs/NRPs. The axonal isolates are depleted of cell body (MAP2 and c-Jun) and glial (GFAP) mRNAs, but contain the known axonal transcript β-actin.

The studies cited above confirmed that factors expressed and secreted by the GRPs and GRPs/NRPs can support growth of host axons and set the stage for the analysis of associated gene expression control mechanisms in the coculture system. Trophic factors, cAMP signaling, and injury are known to increase neurite growth and axon regeneration from DRG neurons. These stimuli share an ability to change the gene expression program in the DRGs, with each increasing expression of several growth-associated gene products through transcription (Smith and Skene, 1997; Neumann et al., 2002; Ma et al., 2014; Chandran et al., 2016). Transport of mRNAs into axons with localized generation of new proteins has been linked to axonal growth both in cultured neurons and *in vivo* (Perry and Fainzilber, 2014), and increasing the transport of mRNAs encoding regeneration-associated genes into axons has been shown to increase axon growth (Donnelly et al., 2013). Thus, we devised a system to expose cultures of adult DRG neurons to the GRPs/NRPs where we could uniquely assess mRNA levels in both the DRG cell bodies and their axons. We modified the culture system that had been developed for quantitatively assessing axonal mRNAs in the DRG neurons using RT-PCR methods (Willis et al., 2005) to allow for coculture of DRGs and GRPs/NRPs (Fig. 3*A*). This provided a system where the DRG neurites were exposed to the environment of the GRPs/NRPs, but could be physically separated for isolation of both cell body and neurite compartments (Fig. 3*C*,*D*).

**Figure 4.**
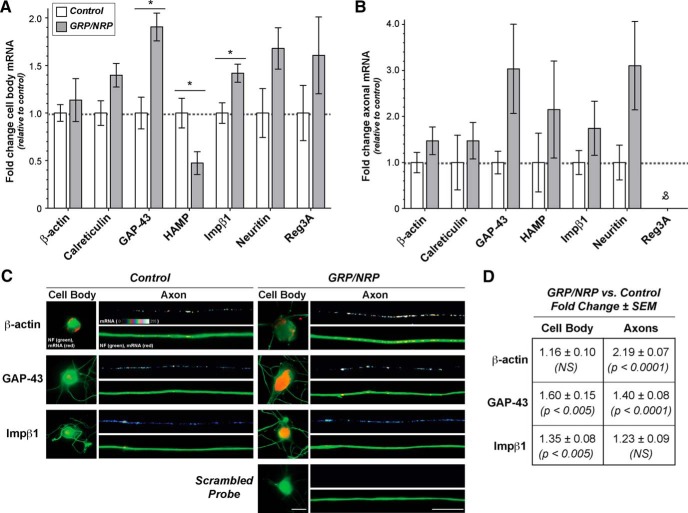
Exposure to GRPs/NRPs alters the levels of growth-associated mRNA cell bodies and axons of adult DRG neurons. ***A***, ***B***, The modified coculture system from Figure 3 was used for analyses of RNA levels from the explanted ganglia (“cell body RNA”) and their extended axons (“axonal RNA”). Data from RT-ddPCR analyses with cell body and axonal RNA preparations for known axonal mRNAs are shown in ***A*** and ***B***, respectively. Values are shown for the NRP/GRP coculture relative to control samples, as indicated, ±SD for biological replicates (*N* = 4; *p* ≤ 0.05 for indicated columns; &no positive droplets were detected for Reg3a amplification despite highest template input levels). ***C***, Representative FISH/IF images of cell bodies and axon shafts are shown for indicated mRNAs for dissociated DRGs cultured under control or GRP/NRP-exposed conditions. For these studies, DRGs were cultured on coverslips over a bed of GRP/NRP cells. Image pairs for control and GRP/NRP conditions are exposure matched, and axonal mRNA signals are shown as a spectral intensity in the upper rows of axon image sequences and cell body, and lower axon panels show merged images for mRNA and NF. Scale bars: cell body panels, 25 µm; axon panels, 10 µm. ***D***, Quantification of axonal FISH signal intensities from matched exposure images are shown for GRP/NRP coculture vs control cultures as the fold change ± SEM (*n* > 50 for axons and *n* > 25 for cell bodies in three culture preparations; *p* values are from ANOVA with Tukey *post hoc* analyses).

Previous work has pointed to the axonal nature of the neurites extended from DRG neurons in culture, with plus-ended microtubule polarity, accumulation of axonal proteins, and exclusion of the somatodendritic MAP2 mRNA and protein (Baas et al., 1987; Smith and Skene, 1997; Zheng et al., 2001; Vuppalanchi et al., 2010). Thus, we will herein refer to the lower membrane surface as an “axonal preparation” and upper membrane surface as a “cell body preparation”; although it should be noted that the explants from the upper surface also contain Schwann cells, satellite cells, and axonal processes. Using RT-PCR, we were able to confirm the isolation of a highly enriched complement of RNA from the axonal compartments, with near exclusion of the cell body c-Jun and MAP2 mRNAs as well as GFAP mRNA that is selectively expressed by Schwann cells in these cultures (Fig. 3*E*; Zheng et al., 2001; Willis et al., 2007). We advanced to RT-ddPCR to quantitate levels of different mRNAs from cell body and axonal compartments of the DRG cultures, comparing DRG only with DRG plus GRP/NRP cocultures. Looking at mRNA levels in the cell body compartment, coculture with GRP/NRP causes a significant increase in GAP-43 and Importin β1 mRNA levels (Fig. 4*A*). Neuritin and Reg3A mRNAs also showed increased levels in the cell body compartment of the DRGs cocultured with GRPs/NRPs, but this did not reach significance.

Axonal mRNA levels changed comparing the DRGs cocultured with GRPs/NRPs to standard DRG cultures. These axonal RNA analyses showed high variability between preparations, and, although none of these axonal mRNA changes reached statistical significance, trends were apparent. Contrasting the changes in axonal mRNA to the cell body mRNA levels suggests post-transcriptional regulation of the axonal mRNA content. Levels of GAP-43, Importin β1, and Neuritin mRNAs increased in the axons cocultured with GRPs/NRPs, paralleling the increased cell body levels of these mRNAs (Fig. 4*B*). On the other hand, axonal hepcidin antimicrobial peptide (HAMP) mRNA, which was decreased in the cell body compartment preparations of the DRGs cocultured with GRPs/NRPs (Fig. 4*A*), was increased in the axonal compartment (Fig. 4*B*). Finally, these changes were not universal, in that Reg3a mRNA was not detected in the axons yet it was clearly amplified from the cell body compartments of both the control DRGs and DRGs cocultured with GRPs/NRPs (Fig. 4*A*,*B*). Transcriptional increases of Reg3A and HAMP mRNAs were previously shown in L4/5 DRGs following an *in vivo* sciatic nerve crush, and both mRNAs show increased localization to regenerating axons *in vitro* and *in vivo* after sciatic nerve crush (Kalinski et al., 2015). Together, these data suggest that the exposure of DRG neurons to progenitor cells can trigger changes in levels of specific mRNAs through both transcriptional and post-transcriptional mechanisms.

Considering the variability in the above RT-ddPCR analyses of DRG axons, we performed quantitative FISH for endogenous β-actin, GAP-43, and Importin β1 mRNAs. We used Stellaris FISH probes whose signals in axonal processes have proven highly reproducible in previous studies (Spillane et al., 2013; Perry et al., 2016). To be certain that FISH signals were from the neurons rather than GRPs/NRPs, we cultured dissociated DRGs on coverslips laid over a bed of GRPs/NRPs. FISH images showed an increase in β-actin and GAP-43 mRNAs but not Importin β1 mRNA in axons of DRGs cocultured with GRPs/NRPs compared to control cultures (Fig. 4*C*). Quantification across multiple culture preparations showed a significant increase in axonal β-actin and GAP-43 mRNAs with GRP/NRP cocultures versus control cultures (Fig. 4*D*). Cell body levels of both Importin β1 and GAP-43 mRNAs also increased significantly in DRGs cocultured with the GRPs/NRPs compared with control (Fig. 4*D*). The increased FISH signals for GAP-43 and Importin β1 mRNAs in the cell body and GAP-43 mRNA in axons are consistent with those seen by RT-ddPCR above, but the increased axonal FISH signals for β-actin mRNA conflict with the data in Figure 4*B*, where no significant change in axonal β-actin mRNA levels was detected.

### Coculture with GRPs/NRPs increases axonal mRNA localization in adult DRG neurons

**Figure 5.**
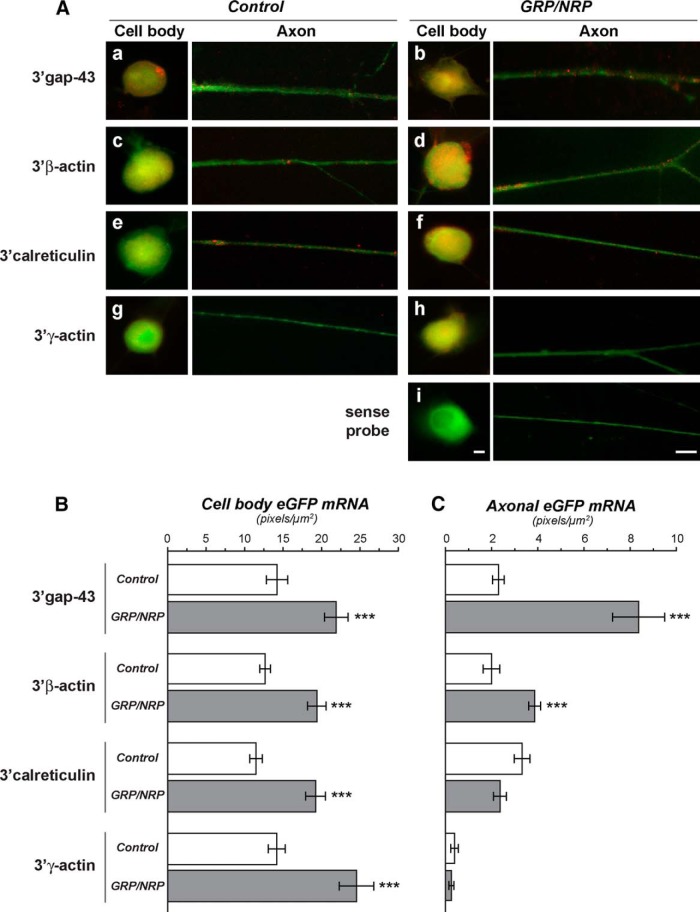
Alterations in axonal mRNA levels with GRP/NRP coculture is conferred by axonal mRNA UTRs. ***Aa–i***, Representative fluorescent images for eGFP mRNA (red) and βIII-tubulin protein (green) in cell body and distal axon shaft of dissociated DRG cultures transfected with eGFP^MYR^ plasmids with 3'UTRs of rat GAP43 (***a***, ***b***), β-actin (***c***, ***d***), CALR (***e***, ***f***), and γ-actin mRNAs (***g***, ***h***). ***i***, Images for sense eGFP probe represent the 3'β-actin construct. Left-hand columns of axon and cell body images show control DRG cultures, and right-hand columns show DRGs cocultured with GRPs/NRPs. All image pairs are exposure matched (control vs GRP/NRP coculture axon and control vs GRP/NRP coculture cell body). As previously published, axonal GFP mRNA is not seen for the construct with 3'UTR of γ-actin, but axonal signals are seen for GAP43, calreticulin, and β-actin 3'UTR constructs (Willis et al., 2007; Vuppalanchi et al., 2010; Yoo et al., 2013). ***B***, ***C***, Quantitation of axonal and cell body GFP mRNA intensities across multiple transfection experiments for DRGs with or without GRPs/NRPs are shown as average signal intensities ± SEM (*n* ≥ 30 processes over three independent transfections and cultures; ***p ≤ 0.001 by Student’s *t* test). Scale bars, 20 µm.

With the conflicting results seen between RT-ddPCR from purified axons versus quantitative FISH for axonal β-actin mRNA from the intact DRG cultures, we used transfection with constructs encoding eGFP^MYR^ with axonally localizing 3'UTRs of β-actin, GAP-43, and calreticulin mRNAs (Willis et al., 2007; Vuppalanchi et al., 2010; Yoo et al., 2013) to directly test for transport of mRNAs into axons. We reasoned that this would allow us to determine whether coculture with GRPs/NRPs could affect axonal levels of mRNAs through 3'UTR motifs that are known to be necessary and sufficient for axonal localization in adult sensory neurons. For this, dissociated DRGs were transfected with eGFP^MYR^ 3'β-actin, eGFP^MYR^3'gap-43, or eGFP^MYR^3'calr plasmids (Willis et al., 2007; Donnelly et al., 2013). eGFP^MYR^ with the nonlocalizing 3'UTR of γ-actin mRNA (eGFP^MYR^3'γ-actin) was used as a control for possible nonspecific diffusion of mRNA (Willis et al., 2007). After transfection, the DRGs were plated directly onto a bed of rat GRPs/NRPs or alone and cultured in GRP–basal media for 3 d. Consistent with previous findings (Donnelly et al., 2013), DRGs transfected with eGFP^MYR^3'γ-actin showed no axonal GFP mRNA signals by FISH, both with control culture and GRP/NRP coculture conditions (Fig. 5*A*). Axonal eGFP mRNA signals were easily detected for the eGFP^MYR^3'β-actin, eGFP^MYR^3'gap43, and eGFP^MYR^3'calr expressing DRGs under both conditions. However, the intensity of the axonal eGFP^MYR^ mRNA signals was clearly different between the growth conditions. Axonal RNA signals for eGFP^MYR^3'β-actin and eGFP^MYR^3'gap43 appeared higher in the DRGs cocultured with GRPs/NRPs (Fig. 5*A*).

Quantification of the axonal FISH signals for eGFP mRNA across biological replicates showed a significant increase in eGFP^MYR^3'β-actin and eGFP^MYR^3'gap43 mRNAs when DRGs were cocultured with GRPs/NRPs (Fig. 5*B*). On the other hand, the axonal levels of eGFP^MYR^3'calr showed no significant change in the GRP/NRP-exposed DRGs (Fig. 5*B*). Differences in transcription rates and stability of the reporter mRNAs between the eGFP^MYR^3'β-actin, eGFP^MYR^3'gap43, and eGFP^MYR^3'calr constructs could explain the altered axonal mRNA levels in the DRGs cocultured with GRPs/NRPs. Quantification of the eGFP mRNAs in the neuronal cell bodies showed a significant increase in FISH signaling for all four constructs, including eGFP^MYR^3'γ-actin, which showed no localization into the axons, and the eGFP^MYR^3'calr, which showed no difference in axonal levels comparing control DRGs to those cocultured with GRPs/NRPs (Fig. 5*C*). Since each plasmid uses the same sequence for CMV promoter and 5'UTR, the increased axonal levels of eGFP mRNA with 3'UTRs of β-actin and GAP43 mRNAs are the result of post-transcriptional regulation driven by differences in the 3'UTRs of these mRNAs.

## Discussion

Transplantation of progenitor cells has been pursued as an experimental neural repair strategy for many different neurological conditions, ranging from neurodegenerative diseases to traumatic injury (Lu et al., 2014; Tso and McKinnon, 2015; Lane et al., 2016). These approaches hold high promise for translation to clinical settings. Much experimental effort has been focused on how the progenitor cells integrate into the nervous system and develop the morphologies and functions of their more mature counterparts. These are obviously critical steps for the utility of these cell-based therapies in any neural replacement strategy. However, host neurons must also interact with the grafted progenitors, and these interactions have received relatively less attention. We have used a coculture approach to reconstitute the minimal cellular components for host neuron and progenitor cell interactions to begin to explore the effects of progenitor cell grafts on host neurons. Both GRPs and the combination of GRPs/NRPs increased axonal outgrowth from adult DRG neurons in coculture. The DRGs would comprise a portion of the ascending axons that previously showed growth into grafts of GRP and GRP/NRP cells in the injured spinal cord (Bonner et al., 2011). The neurite outgrowth from adult DRG neurons was surprisingly greater exposing the cells to conditioned medium from the GRPs than the coculture condition (Fig. 1). This suggests that cell–cell contact between axons and GRPs or the extracellular matrix generated by the GRPs could limit axon outgrowth. The mixture of GRPs/NRPs for coculture had less of a growth-promoting effect than GRPs alone, so the presence of NRPs may also limit the growth-promoting effects of GRPs.

It should be noted that the increased axonal localization of β-actin mRNA seen by FISH for the endogenous mRNA and transfected GFP^MYR^ with 3'UTR of β-actin mRNA was not seen by RT-ddPCR from isolated axons (compare Figs. 4*C*,*D*, 5 vs Fig. 4*B* ). There was substantial variability with the RT-ddPCR analyses. The axonal isolates for PCR included much longer lengths of axons than could be considered by the FISH analyses that concentrated on distal axonal segments where we could exclude any diffusion from the cell body. This difference in the sites of the axons analyzed could account for the discrepancy of RT-ddPCR and FISH data for β-actin mRNA. The possibility that the FISH studies represent mRNAs recruited into the distal axon from more proximal axon segments bears some consideration; as discussed below, there is previous evidence for the recruitment of β-actin mRNA by trophic factors (Willis et al., 2007).

The increase in axon growth from DRGs seen with the GRP/NRP cocultures was accompanied by changes in the expression of regeneration-associated genes and axonal localization of some of these mRNAs. These changes in gene expression through transcriptional and post-transcriptional regulation may underlie the growth-promoting effects of the progenitor cells upon the DRG neurons. Interestingly, the growth-promoting effects of the precursor cells were also seen in injury-conditioned neurons, which were shown to have higher growth capacity when initially cultured (Smith and Skene, 1997). However, the changes in mRNA levels in the cell bodies and axons of naive DRGs cocultured with GRPs/NRPs did not fully replicate the changes in gene expression previously published for injury-conditioned DRG neurons. Increased expression and axonal localization of GAP-43 mRNA is seen in both the naive DRG-GRP/NRP coculture (Fig. 4) and L4/5 DRGs after sciatic nerve injury (Van der Zee et al., 1989; Yoo et al., 2013). Neuronal Reg3A mRNA was not significantly changed, and HAMP mRNA showed a significant decrease in the naive DRG-GRP/NRP coculture (Fig. 4), while these mRNAs increase by ∼7- and ∼1800-fold, respectively, in L4/5 DRGs after sciatic nerve crush (Kalinski et al., 2015). HAMP and Reg3A mRNAs also increased in axons of L4/5 DRGs (by ∼5-fold and ∼15-fold, respectively) following sciatic nerve crush injury (Kalinski et al., 2015), but Reg3A mRNA was not detected in the axons of the naive DRG-GRP/NRP cocultures. However, the HAMP mRNA increase in the axons did not reach significance. These observations suggest that the axon growth-promoting effects of injury conditioning have some mechanisms that are distinct from the exposure to NPCs studied here. However, it should be noted that molecular responses of the DRGs cocultured with GRPs could be different than in the DRG-GRP/NRP cocultures that we used to test gene expression; so we cannot rule out an effect on Reg3A mRNA from GRPs based on the data presented here.

During development, proteins generated from axonal mRNAs have been shown to contribute to growth cone responses from both positive and negative guidance cues (Hengst and Jaffrey, 2007; Jung et al., 2011). This was initially seen in cultured neurons, but *in vivo* studies point to functions of axonally generated proteins during development of the spinal cord (Brittis et al., 2002; Tcherkezian et al., 2010; Colak et al., 2013; Donnelly et al., 2013). Although the possibility that mature neurons synthesize proteins in axons was initially doubted (Steward, 2002), translation has been demonstrated in axons of adult DRG neurons in culture and *in vivo* (Perry and Fainzilber, 2014). Ascending axons in the spinal cord contain mRNAs and translational machinery when they regenerate into a peripheral nerve graft, with some mRNAs showing comparable levels to regenerating sciatic nerve axons (Kalinski et al., 2015). These peripheral nerve grafts contain axons from dorsal horn interneurons as well as centrally projecting propriospinal axons from the DRGs (Sachdeva et al., 2016). With the shifts in axonal mRNA levels shown here when DRGs are cocultured with GRPs/NRPs, it will be a high priority to determine whether GRP/NRP or other stem cell grafts increase axonal mRNA transport in host spinal cord axons *in vivo*. In this study, we did not note any differences in axon outgrowth or reporter mRNA localization with those of axons based on neuron cell body diameters that define nociceptive, mechanoreceptive, and proprioceptive neurons. So, the activities of the factors secreted by the progenitor cells are unlikely to be limited to the ascending propriospinal axons.

Post-transcriptional regulation of gene expression, including the subcellular mRNA localization studied here, has increasingly been implicated in neuronal development and function. Decreased axonal levels of β-actin and GAP-43 mRNAs in mouse PNS axons were shown to delay nerve regeneration (Donnelly et al., 2011). Conversely, increasing axonal localization of GAP-43 mRNA in embryonic chick spinal cord triggered longer axon growth, while increasing axonal localization of β-actin mRNA under these conditions caused increased branching of axons (Donnelly et al., 2013). It is intriguing to speculate that the changes in axonal localization of mRNAs with the GRP/NRP coculture system used here contribute to the increased axon growth seen with the coculture as well as the possibility for altering host axon growth *in vivo*. Of course, alterations in the survival of axonal mRNAs could account for the changes in axonal levels of endogenous and/or transfected reporter mRNAs (Figs. 4, 5, respectively). Regardless, the changes in reporter mRNA levels in the axons shown here (Fig. 5) clearly indicate a post-transcriptional mechanism through the 3'UTRs of β-actin, GAP43, and calreticulin mRNAs, which accounts for the effects of the GRP/NRP coculture on the reporter mRNAs.

Previous studies have shown that axonal mRNA transport can be regulated by neurotropic factors and injury conditioning in cultured DRG neurons (Willis et al., 2005, 2007; Cosker et al., 2013). Most *cis*-acting elements that target mRNAs for subcellular localization have been found in UTRs of the transported mRNAs, particularly the 3'UTRs (Gomes et al., 2014). RNA-binding proteins that are needed for the transport of these mRNAs interact with these RNA structures. For example, mouse and rat β-actin mRNAs contain a 54 nucleotide (nt) sequence that is recognized by ZBP1 protein; the resulting RNA–protein complex interacts with motor proteins for axonal and dendritic localization (Zhang et al., 2001; Eom et al., 2003). For GAP43 mRNA, an ∼40 nt AU-rich element in its 3'UTR is both necessary and sufficient for axonal localization (Yoo et al., 2013). The Elav-like protein HuD binds to this region of GAP43 mRNA. ZBP1 protein also contributes to GAP43 mRNA localization in rat and mouse DRG axons (Donnelly et al., 2011; Yoo et al., 2013). Since both endogenous GAP-43 and β-actin as well as eGFP^MYR^3'β-actin and eGFP^MYR^3'gap43 mRNAs showed increased localization in DRG axons in the presence of GRPs/NRPs (Fig. 5), the intracellular effects of exposure to GRPs/NRPs could increase levels or the RNA-binding activities of ZBP1, HuD, and/or other proteins needed for axonal transport of GAP43 and β-actin mRNAs. Further, with the axonal levels of eGFP^MYR^3'calr decreasing with the GRP/NRP coculture, one may surmise that stimuli derived from the decrease in GRP/NRP levels or the activity of proteins needed for axonal localization of calreticulin mRNA.

Secreted neurotrophic and/or neurotropic factors are appealing candidates for the growth-promoting effects of the GRPs/NRPs. β-Actin has been shown to accumulate at localized sources of neurotrophins (Willis et al., 2007), and even the application of neurotrophins along one side of a growth cone can result in an asymmetrical redistribution of β-actin mRNA in distal growth cones (Yao et al., 2006). The secretome has been characterized for some stem cell populations (for review, see Salgado et al., 2015; Shoemaker and Kornblum, 2016). Bone marrow stromal cells (MSCs) secrete a number of neurotrophic factors in culture (Neuhuber et al., 2005; Nakano et al., 2010) and when grafted into the CNS (Teixeira et al., 2013; Jablonska et al., 2015). Similar to the effects of GRPs/NRPs, conditioned medium from bone marrow-derived mesenchymal cells was shown to increase axonal growth from DRG explant cultures (Neuhuber et al., 2005), pointing to the possibility of soluble factors as trophic agents for host axon growth. However, the MSCs required direct contact between neurons and MSCs for DRG axons to extend on nonpermissive substrates (Wright et al., 2007), while the GRP-conditioned medium by itself was shown to support DRG axon growth on nonpermissive CSPGs (Ketschek et al., 2012). The differential growth effects that we see with conditioned medium from the GRPs alone versus a GRP/NRP combination may reflect some restriction of the growth-promoting effects of the GRPs by the presence of NRPs. This could provide a selective advantage *in vivo* by facilitating interactions between host axons and grafted progenitors. Thus, it will be of interest to determine whether GRPs versus GRPs/NRPs differentially alters the mRNA populations of host axons.

In summary, our data indicate that NPCs can impact the growth of host axons as well as change neuronal gene expression at both transcriptional and post-transcriptional levels. With axonal levels of some mRNAs increasing on coculture with NPCs without a corresponding change in the cell body levels of the mRNAs, our studies suggest that exposure to NPCs may directly impact the transport of mRNAs into host axons, thereby increasing the intrinsic capacity for these axons to grow.

## Synthesis

Reviewing Editor: Michael Fainzilber, Weizmann Institute of Science Decisions are customarily a result of the Reviewing Editor and the peer reviewers coming together and discussing their recommendations until a consensus is reached. When revisions are invited, a fact-based synthesis statement explaining their decision and outlining what is needed to prepare a revision will be listed below. The following reviewer(s) agreed to reveal their identity: Antonella Riccio, Valerie Verge

This manuscript reveals a probable mechanism by which neural progenitor cells are able to promote axon growth, namely by driving axonal localization of mRNAs encoding growth-associated proteins. In general these are well thought-out experiments with appropriate controls. There are however some issues which should be addressed, as follows:

(1) The evidence of mRNA transport in axons cannot be based only on a reporter assay. FISH of the endogenous RNAs and western blot and/or immunocytochemistry showing protein levels should also be presented.

(2) It would be very interesting to see what effects the GRPs and GRP/NRPs would have on injury-conditioned sensory neurons. The addition of such experiments would significantly strengthen the paper.

(3) Please clarify the sentence in lines 8-10 of the abstract, which states:

“Conditioned medium from either GRPs or GRP/NRPs similarly increased axon growth from adult DRG neurons, with the GRPs but not GRP/NRPs triggering even greater axon extension from DRGs than the co-culture condition”

From the data it looks like the conditioned medium from the GRPs (210%) was more effective than the GRP/NRP (130%) conditioned medium in driving the neurons to longer individual axon branches - why do the authors describe this as “similarly increasing growth”?. The representative photos show extensive out growth in both in response to CM. How did the overall total outgrowth per neuron compare for the different conditions? Please include this data and also whether there were differences in the actual outgrowth in the DRG only control cultures in the GRP vs. GRP/NRP experiments. With respect to the latter, were the same control cultures used to compare both experimental conditions (i.e. CM from GRP vs GRP/NRP)? Could it be that the shorter single branch length difference might be because only a percentage of the cells (GRPs in the GRP/NRP) were secreting the growth promoting compounds (and not necessarily that one cell type inhibits the production of growth promoting compounds as suggested on line. Is anything known about what the percentage of cells in the mixed GRP/NRP cultures are actually GRP cells?

(4) With respect to the conditioned medium how were bFGF and NT-3 excluded if they were plated in the presence of 10 ng/ml bFGF and then the CM collected 48 hrs later?

(5) Were any differences observed in the male versus female responses?

(6) On line 573 were the GRPs or the NRP/GRPs visualized with DiO stain?

(7) Given that GRPs CM DRG cultures elicited approximately ~80% greater outgrowth than that of the NRP/GRPs CM DRG experiments (~130% vs. ~210%) were the experiments represented by Figs 3&4 done with GRPs cultured on the bottom of the well? This would be an interesting addition.

(8) The author should increase the axonal compartment N to see if any of the changes reach significance (is the NMP35 value of the neurons exposed to GRP/NRPs really not significantly different from control?).

(9) Consider rearranging Figures 3 and 4, since fig.4A is basically a control for Fig.3.

(10) The manuscript would benefit from a more detailed introduction on the role of mRNA localization in axon growth and more generally, neuronal functions.
